# An update on *Leishmania martiniquensis* infections: Transmission, clinical characteristics, and treatment

**DOI:** 10.1016/j.parepi.2024.e00386

**Published:** 2024-10-20

**Authors:** Somayyeh Ahmadi, Maryam Hataminejad, Bahman Rahimi Esboei, Seyed Abdollah Hosseini, Mahdi Fakhar

**Affiliations:** a Student Research Committee, Mazandaran University of Medical Sciences, Sari, Iran; b Toxoplasmosis Research Center, Communicable Diseases Institute, Faculty of Medicine, Mazandaran University of Medical Sciences, Sari, Iran; c Iranian National Registry Center for Lophomoniasis and Toxoplasmosis, Imam Khomeini Hospital, Mazandaran University of Medical Sciences, P. O Box: 48166-33131 Sari, Iran

**Keywords:** Leishmaniasis, *Leishmania martiniquensis*, Amphotericin B, Clinical features, Midges

## Abstract

Leishmaniasis, caused by intracellular protozoa of the *Leishmania* genus, continues to be a global health issue, with approximately 700,000 to 1 million new cases occur annually worldwide. The disease is transmitted via the bite of infected female sand flies of the genus *Phlebotomus*, resulting in a range of symptoms known as cutaneous, mucocutaneous, and visceral leishmaniasis. The species *Leishmania (Mundinia) martiniquensis*, discovered in 1995, has been linked to cases in individuals with HIV, presenting with diverse clinical pictures. Interestingly, biting midges, not sandflies, has proved to serve as its potentially biological vector. This study focuses on understanding the transmission, clinical aspects, and effective treatment of L. *martiniquensis* infections.

A comprehensive search strategy was employed to identify relevant published papers on the epidemiology, transmission, clinical characteristics, and treatment of L. *martiniquensis* up to August 2024. The clinical manifestations encompass localized cutaneous leishmaniasis, disseminated cutaneous leishmaniasis, mucocutaneous leishmaniasis, and visceral leishmaniasis. Leishmaniasis is associated with comorbidities such as inadequate nutrition, population displacement, and reduced immunity. Risk factors for *Leishmania* infection include the presence of domestic animals, age, gender, and environmental factors. Amphotericin B deoxycholate (AmB) is the main treatment. Combination therapy with allicin and andrographolide may reduce AmB side effects. Recent research investigates other treatments including 8-hydroxyquinoline, which works synergistically with AmB against L. *martiniquensis*.

## 1 Introduction

Leishmaniasis is caused by intracellular protozoa of the *Leishmania* genus, a member of the Order Kinetoplastida, Family Trypanosomatidae. Leishmaniasis is spread through the bite of a female phlebotomine sand fly (a 2–3-mm insect vector) that carries metacyclic promastigotes, a transmissible form of the parasite, into the host's skin (Ghazanfar and Malik, 2016). The host's macrophages phagocytose the parasites, and the promastigote transforms into the amastigote, an immotile, obligatory intracellular stage that allows the parasites to proliferate and spread to other cells (Handman and Bullen, 2002).

The purpose of this study is to focus on *Leishmania (Mundinia) martiniquensis* and the associated risk factors in the transmission and development of the disease, including its host and vector, as well as effective medication treatments for combating this parasite. The search was conducted in four scientific databases such as PubMed, Scopus, ScienceDirect and Google Scholar to find relevant papers up to August 2024. Multiple keywords were used when searching for articles, such as “*Leishmania martiniquensis*” AND “treatment” OR “manifestations” OR “biting midges” OR “vector”.

### 1.1 Taxonomy

**Fig. 1:**
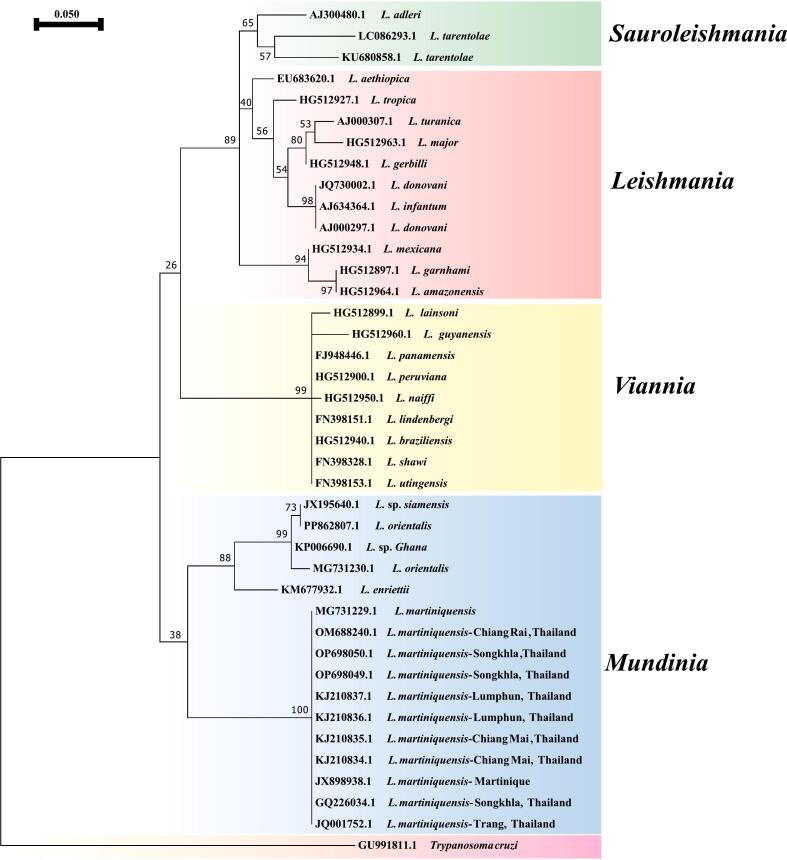
Maximum likelihood phylogenetic tree of the *Leishmania* based on internal transcribed spacer 1 sequences. The evolutionary relationships among diverse species are illustrated by the tree, which includes representatives from different *Leishmania* subgenera (*Mundinia*, *Viannia*, *Leishmania*, and *Sauroleishmania*). The nodes are provided with bootstrap values from 1000 replicates. *Trypanosoma cruzi* is classified as an outgroup.

The genus *Leishmania* is classified into four primary subgenera based on morphological, biochemical, and molecular characteristics. Each subgenus encompasses species with distinct ecological niches and pathogenic profiles. Subgenus *Leishmania* includes species such as *L. major* and *L. tropica*, which are primarily responsible for cutaneous leishmaniasis (CL) in the Old World. These species are transmitted by sandflies of the genus *Phlebotomus*. The clinical manifestations can range from localized skin lesions to more severe systemic forms, depending on the host's immune response and other factors. The subgenus *Sauroleishmania* is notable for its association with reptilian hosts. Species such as *Leishmania tarentolae* primarily infect reptiles and are not typically pathogenic to humans. However, their role in the ecosystem as potential reservoirs for zoonotic transmission is of increasing interest, particularly in light of changing environmental conditions that may facilitate cross-species transmission. The subgenus *Viannia* Predominantly found in South America, this subgenus includes species like *L. braziliensis*, which is known for causing both cutaneous and mucocutaneous leishmaniasis (MCL). The transmission dynamics are closely linked to specific vectors from the genus *Lutzomyia*. Understanding the epidemiology of this subgenus is crucial for public health strategies in endemic regions. The recently identified subgenus *Mundinia* includes species such as L. *martiniquensis*, which has been documented in the Caribbean and is associated with cutaneous leishmaniasis. Research on this subgenus is still evolving, but it highlights the diversity within the genus and the need for continued exploration of its clinical significance and transmission dynamics (Kwakye-Nuako et al., 2023; Sereno, 2019). Its taxonomical position was defined in 2002, its nomenclature was designated in 2014. It is a species within the L. *enriettii* complex that causes both cutaneous and visceral leishmaniasis (VL) in humans by utilizing isoenzyme analysis, sequencing of the 18S ribosomal RNA gene, and partial sequencing of the major subunit genes of RNA polymerase II and DNA polymerase alpha, the taxonomical level of the parasites causing these two cases (MAR1 and MAR2) was identified (Noyes et al., 2002). *Leishmania martiniquensis* was classified as a member of the zymodeme MON-229 (Leelayoova et al., 2017b). The zymodeme in question was formerly categorized as belonging to the *L. siamensis* lineage. However, subsequent investigation has revealed that it is, in fact, a separate and unique species known as L. *martiniquensis* (Desbois et al., 2014; Leelayoova et al., 2017b). As shown in Fig. 1, a phylogenetic tree constructed using the internal transcribed spacer 1 gene sequence effectively distinguishes L. *martiniquensis* from other *Leishmania* species.

### 1.2 Epidemiology

Over 1 billion persons are now living in regions where leishmaniasis is prevalent and are vulnerable to contracting the disease. Around 30,000 new cases of VL and over 1 million new cases of CL occur each year. It could be mentioned that most parasite-infected people never show symptoms (WHO, Jun 12, 2024)*.* It could be mentioned that most parasite-infected people never show symptoms. Thus, leishmaniasis is the illness caused by a *Leishmania* infection, not the parasite itself (Alvar et al., 2012; Borges et al., 2022; Kindie et al., 2023; Ruiz-Postigo et al., 2021). *Leishmania martiniquensis* was first identified in 1995 (Dedet et al., 1995) in an HIV-positive patient with scattered nodular CL in Martinique, a French island in the Caribbean (Desbois et al., 2014). Later, two further cases including the isolation of the parasites and their identification as monoxenous trypanosomatids by isoenzyme electrophoresis were reported parasite can also infect humans in island of Martinique (Boisseau-Garsaud et al., 2000; Dedet et al., 1995). Subsequently, it has been shown that the Switzerland(Lobsiger et al., 2010a; Müller et al., 2009). *Leishmania martiniquensis* has recently been documented in South America, specifically in southeast Brazil (Mendes Junior et al., 2023). This suggests that the parasite has a broader geographic range than was previously recognized. Moreover, *L. martiniquensis* has a broad geographic range and may infect many mammalian hosts, suggesting that it has a well-established cycle of transmission between animals and humans.

*Leishmania martiniquensis* is classified as a zoonotic parasite, capable of infecting both humans and other animal hosts. Besides humans, *L. martiniquensis* has been observed infecting cows in Switzerland (Lobsiger et al., 2010a) and horses in Germany (Müller et al., 2009), and Florida (Reuss et al., 2012b). For instance, a study reported the detection of L. *martiniquensis* in a mare in Brazil (Mendes et al., 2023a), which exhibited natural healing of skin lesions. Therefore, we could conclude that horses could potentially serve as reservoirs for this parasite species (Mendes et al., 2023b).

**Fig. 2:**
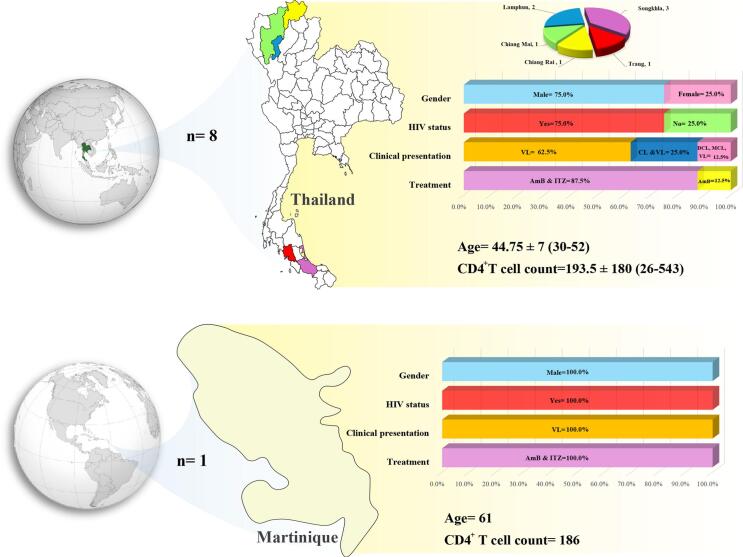
Global distribution of proven leishmaniasis caused by *Leishmania martiniquensis.*

Some differences between L. *martiniquensis*, L. *donovani* and *L. infantum* are included in Table 1 (Fraihi et al., 2017; Ghouse Peer et al., 2024; Hajjaran et al., 2021; Leelayoova et al., 2017b; Preativatanyou et al., 2023; Seblova et al., 2013; Songumpai et al., 2022b; Steverding, 2017; Sunantaraporn et al., 2021; Tabbabi, 2019). The life cycle of L. *matriquinensis* is similar to that of other *Leishmania* species, as it involves two primary morphological forms. It encompasses interactions among the parasite, sandfly vector, and mammalian host. In the sandfly, amastigotes derived from an infected blood meal differentiate into procyclic promastigotes within the midgut, as well as nectomonad, leptomonad, haptomonad, or metacyclic promastigotes. In the mammalian host, injected metacyclic promastigotes convert into amastigotes, resulting in tissue injury and inflammation. The cycle persists when an additional sandfly feeds on the afflicted host (Gossage et al., 2003; Salloum et al., 2021). The global distribution of proven leishmaniasis caused by L. *martiniquensis* is illustrated in Fig. 2.

**Table 1:** A comparison of some differences between Leishmania martiniquensis, Leishmania donovani, and Leishmania infantum.

	Leishmania martiniquensis	Leishmania donovani	Leishmania infantum
Animal reservoir	Black rats	Without any nonhuman reservoir	Dog species of Canis familiaris
Vector	*S. gemmea*, *S.barraudi*, *S. khawi*, *P. Stantoni* *C. mahasarakhamense, C. peregrinus, C. oxystoma,* *C. huffi, C. fordae, C. fulvus*	*P. alexandri*, *P. orientalis, P. celiae* *P. martini, P. argentipes*	*P. perniciosus, P. perniciosus, P. longicupis, P.langeroni, P. perfiliewi, P. ariasi, P. galilaeus,* *P. syriacus, P.tobbi*, *P. halepensis*
Geographical distribution humans and animals	Martinique Island, Caribbean Island, Central Europe, Florida, Thailand, and Myanmar, livestock in Europe and the USA, horses and cattle in Europe and horses in North America	Central Africa, South Asia, Middle East, India, China	Mediterranean countries (North Africa and Europe), Southeast Europe, Middle East, Central Asia, North, Central and South America (Mexico, Venezuela, Brazil, Bolivia)

S=Sergentomyia; P=Phlebotomus; C = Culicoides.

### 1.3 Clinical manifestations

Among the three primary clinical manifestations of leishmaniasis, the most severe form, known as VL or kala-azar, is characterized by parasite proliferation in the reticuloendothelial system, primarily impacting the bone marrow, liver, spleen, and lymph nodes. VL can result in a variety of symptoms, ranging from nothing at all to death (Banuls et al., 2011; Belo et al., 2014). The parasite species and host immunity influence the symptoms (Alemayehu et al., 2016; Banuls et al., 2011; Cota et al., 2011). It is possible to see one or more of the following clinical characteristics: anemia, leucopenia, thrombocytopenia, weight loss, hepatomegaly, and hypergammaglobulinemia. The confirmation of the parasite detection needs to be done either by microscopic inspection or by PCR assay utilizing any clinical samples, such as lymph nodes, saliva, buffy coat, bone marrow aspirates, or other biopsy samples (Sriwongpan et al., 2021).

According to studies, *Leishmania* DNA is already present in infected people's saliva before it can be detected in blood samples. Evidence showed, to the early detection of *Leishmania* DNA in saliva samples, Loop-mediated isothermal amplification (LAMP) has proven to be an effective approach (Nzelu et al., 2019). For example, prior to finding L. *martiniquensis* DNA in blood samples, researchers in a paper published in BMC Infectious Diseases found it in the saliva of two HIV-positive individuals in Thailand. For a period of two months, the patients' blood and saliva samples were successively obtained by the researchers. It was discovered that *Leishmania* DNA could only be found in saliva samples, and that the detection of the DNA in blood took place up to two months earlier. According to this, saliva may be a helpful indicator for identifying *Leishmania* infection early on, particularly in asymptomatic cases. In addition, the researchers showed that following treatment, the amounts of *Leishmania* DNA in saliva decreased suggesting that saliva could be utilized for monitoring treatment response. Saliva sample collection is a feasible strategy for disease surveillance in endemic areas since it is a non-invasive technique appropriate for both young people and the elderly (Siriyasatien et al., 2016).

Nevertheless, the findings of Jundang et al.'s investigation, which studied *Leishmania* infection in HIV patients in Thailand, varied from the previous report. The present study comprised the analysis of 56 persons who were recognized as positive cases of *Leishmania* infection utilizing several diagnostic techniques, including DAT and nPCR tests. Notably, patients had varying outcomes when subjected to different diagnostic techniques. Some individuals tested positive only by nPCR buffy coat or saliva nPCR technique. Actually, 9 cases provided positive results for *Leishmania* infection only through nPCR-buffy coat, whereas 6 cases showed positive results only through nPCR-saliva (Jundang et al., 2024). The variations in detection rates may be attributed to the sensitivity and specificity of PCR tests in various kinds of samples (Carranza-Tamayo et al., 2009; Manomat et al., 2017b; Pandey et al., 2018). The primary cell component of the buffy coat samples is that of *Leishmania*-infected macrophages, which might provide a higher amount of *Leishmania* DNA for identification. Saliva samples, on the other hand, may contain less *Leishmania* DNA, which could lead to a reduced detection rate. *Leishmania* DNA may be present and detectable in saliva samples and buffy coats depending on the dynamics of *Leishmania* replication and shedding in various bodily areas. Variable detection rates may also be attributed to variations in the timing of sample collection and the individual's stage of *Leishmania* infection. In buffy coat samples, *leishmania* DNA may be easier to find in the initial phases of infection. *Leishmania* DNA detection in buffy coat samples could decrease while the disease develops. On the other hand, DNA detection in reflective samples may rise, indicating continued mucosal or oral shedding (Pandey et al., 2018). Furthermore, nPCR-saliva and nPCR-buffy coat both revealed *Leishmania* DNA, indicating that the infection had progressed to other parts of the body.

Localized cutaneous leishmaniasis (LCL) is the term used to describe the clinical presentation of CL in immunocompetent patients caused by L. *martiniquensis*, characterized by lesions confined to the skin's inoculation site. Nevertheless, a significant proportion of skin lesions in HIV/AIDS patients may be single, numerous nodular, or vast popular in appearance. The lesion may extend beyond the initial site on the face, ears (including the helix and antihelix of the pinna), hands, fingers, elbows, forearm's extensor surface, trunk, and lower extremities, resulting in disseminated diffused cutaneous leishmaniasis (DCL). It can also appear as multiple non-ulcerative nodules, popular with ulcerative lesions, or as chronic, widespread fibrotic lesions (Chiewchanvit et al., 2015; Chusri et al., 2012; Noppakun et al., 2014; Phumee et al., 2013). Generally, clinical signs and symptoms of CL include localized, widespread, or disseminated cutaneous papules, plaques, and nodules with core ulceration that are usually encircled by roll-edged borders.

Furthermore, severe CL in the head region has the potential to spread to the “leonine face” (McGwire and Satoskar, 2014; Pearson and Sousa, 1996). The confirmation of the parasite detection needs to be done either by PCR test using the skin biopsies or by microscopic analysis of the biopsies (Sriwongpan et al., 2021).

Asymptomatic *Leishmania* infection is characterized by positive results from direct agglutination test (DAT) or PCR assays but no symptoms of CL/VL (Piyaraj et al., 2024; Sriwongpan et al., 2021).

Mucocutaneous Leishmaniasis is a condition characterized by ulcerations in mucosal tissue, most commonly affecting the nasal mucosa. However, it can also occasionally impact the lips, soft palate, pharynx, and larynx (Chappuis et al., 2007; Reithinger et al., 2007).

For example, VL caused by L. *martiniquensis* was first reported in the Caribbean in 2015 by Liautaud and et.et al. The patient was a 61-year-old man infected with the HIV virus. Hepatosplenomegaly, fatigue, and anemia were the disease's main symptoms (Liautaud et al., 2015). In 2022, Srivarasat et al. reported a case of autochthonous multiform leishmaniasis caused by L. *martiniquensis*, which included DCL, MCL, and VL (Srivarasat et al., 2022). It's important to note that studies have only identified one case thus far, and it is not a typical MCL compared to other cases caused by *Leishmania* spp.

### 1.4 Potential vectors

Phlebotomines are the single or primary carriers of *Leishmania*. The three primary genera of sandflies in the Phlebotomidae family are *Lutzomyia* within the New World, *Phlebotomus* and *Sergentomyia* within the Old World. There are eleven subgenera in the genus *Phlebotomus*, which are found throughout Asia, Africa, and Europe.

Nevertheless, *leishmania* DNA has been discovered in multiple species of biting midges, *Culicoides* spp., like *C. imicola* and *C. circumscriptus*, even though these insects are not currently thought to be *Leishmania* parasite vectors (Slama et al., 2014). A study examined the growth of L. *infantum* in a controlled population of *Culicoides nubeculosus* in a laboratory setting. However, it was discovered that L. *infantum* was unable to fully mature in the midgut of *C. nubeculosus* (Seblova et al., 2014). In another study, Seblova et al. (2015) showed that L. *enriettii* parasites can reach the advanced stage of infection in *C. sonorensis*. They also found that these biting midges may get contaminated by feeding on domestic guinea pigs (*Cavis porcellus*) that are infected with L. *enriettii* (Seblova et al., 2015).

In addition, a study examined the growth of *Leishmania (Munidia) orientalis* in two experimental carriers, *Lutzomyia longipalpis* and *C. sonorensis*. On the first day post-infection by the parasites, the amastigotes transformed into procyclic promastigotes in the abdominal midgut.

During the second day of the post infected blood meal (PIBM) experiment, nectomonad promastigotes were detected in the abdominal midgut and subsequently moved to the thoracic midgut.

Leptomonad promastigotes were observed at the third day after inoculation in the tissue macrophage culture. Leptomonad promastigotes and metacyclic promastigotes formed clusters around the stomodeal valve starting from day 3 postinfected blood meal (PIBM). These clusters were accompanied by the buildup of a gel-like substance secreted by the promastigotes.

On day 5 PIBM, promastigotes resembling haptomonads were found. By day 7 post-infection, the proportion of metacyclic promastigotes had increased to 23 %. The findings indicate that biting midges could potentially transmit *L. orientalis* (Chanmol et al., 2019).

Currently, a small number of molecular markers, such as cytochrome *b* of mitochondrial DNA, ITS2, and the D8 domain of ribosomal DNA, are utilized in phylogenetic studies and DNA barcoding in addition to morphological features to identify different species of sandflies. Depaquit et al. (2015) used both techniques to identify *Phlebotomus* (*Anaphlebotomus*) *stantoni* that were caught in Southeast Asian nations, including Thailand (Chiang Mai Province), Malaysia, and Vietnam (Depaquit et al., 2015).

Many sand fly species are recognized as naturally occurring carriers of *Leishmania* spp., belonging to the subgenera *Viannia* and *Leishmania* (Cecílio et al., 2022). Becvar and colleagues' study has demonstrated the recognition of certain midge species as potential vectors for L. *martiniquensis*, a member of the subgenus *Mundinia*.

The research has demonstrated that L. *martiniquensis* can effectively colonize at the stomodeal valve, produce a higher percentage of metacyclic forms than sand flies, and can experimentally infect BALB/c mice with bites from *C. sonorensis*. These results have drawn attention to *Culicoides* spp., as possible vectors of the *Mundinia* subgenus members who could be involved in the natural transmission of leishmaniasis. Further experiments and investigations are required to identify *Culicoides* as the natural vectors and to investigate reservoir hosts (Becvar et al., 2021). *Leishmania*'s entire life cycle includes transitioning from one host, which is a mammal, to another, which is a phlebotomine sand fly (Killick-Kendrick, 1990). Before being transmitted to a host during a subsequent blood meal, parasites in various developmental stages (Bates, 2007) must undergo several crucial steps: (1) surviving a proteolytic attack within the female fly's midgut during a blood meal, (2) escaping the peritrophic membrane, (3) inhibiting peristalsis and adhering to the midgut epithelia (*Leishmania*) or the hindgut (*Viannia*, some *Sauroleishmania*), (4) preventing competition from the gut microbiota, and (5) obtaining nutrients for morphogenesis and migrating to the anterior midgut (excluding *Sauroleishmania*) (Sacks and Kamhawi, 2001; Takken et al., 2013).

Kaewmee and colleagues carried out a study to examine and capture live L. *martiniquensis* and L. *orientalis* parasites from naturally infected *Culicoides* biting midges. Identifying these midges as potential natural leishmaniasis vectors was the primary goal, which is an important criterion in determining their involvement. Evidence indicated the occurrence of *Leishmania* parasites in the foregut of midges that were affected. Furthermore, some other findings include: *Culicoides peregrinus* has the ability to transmit leishmaniasis. The presence of L. *martiniquensis* was detected in *C. peregrinus* midges. *Crithidia* spp.*,* were detected in *C. peregrinus* midges as well. Additionally, midges were shown to have a simultaneous infection with L. *martiniquensis* and *Crithidia* spp. Midges infected with L. *martiniquensis* were found to be 2–6 % common, according to their findings (Kaewmee et al., 2023).

### 1.5 Risk factors linked to infection with L. *martiniquensis*

The world's poorest people are affected by this disease; morbidity is linked to inadequate nutrition, population displacement, substandard housing, reduced immunity, and a lack of financial support. Leishmaniasis is linked to human activities that have negative effects on the environment, such as the deforestation of forests, the construction of dams, the establishment of systems of irrigation, and the growth of urban areas.

Findings from the primary research of this type in Thailand to identify potential risk factors for leishmaniasis disease among immunocompetent people included exposure to termite mounds (the Hmong hill tribes typically inhabit colder, higher elevations. Their homes, traditionally built without windows and placed directly on the ground, consist of bricks, wood, or mud walls) (Sriwongpan et al., 2021). All of these factors could increase the likelihood of *Leishmania* transmission because of the housing settings consist that are ideal for vectors, such as moist, dark places to breed and rest during the day and mud walls with holes and fissures (Calderon-Anyosa et al., 2018), being older (leishmaniasis scars are less common in the elderly, however active lesions are slightly more common in this age group (Reithinger et al., 2003), female (females were probably exposed to more vector bites than males because they spent more time at home, where the environment was ideal for vectors to spawn and rest.), having domesticated animals in a housing area, and being in an animal enclosure (the animals serve as a source of blood for the vectors, and the buildup of animal excrement may attract these insects, bringing them closer to humans and raising the danger of a bite (Ryan et al., 2006); (Sriwongpan et al., 2021).

Moreover, when DNA from L. *martiniquensis* was found in the buffy coat of a single black rat that was caught near the vocational school, this investigation verified that black rats may act as a natural animal reservoir of the parasite. Furthermore, it has been documented that L. *martiniquensis* causes zoonotic transmission of CL in horses from German (Muller et al., 2009), cattle in Switzerland (Lobsiger et al., 2010b), and a horse from Florida, USA (Reuss et al., 2012a).

Several investigations have demonstrated that the majority of human cases documented so far have exhibited the clinical features of disseminated and/or VL often in conjunction with HIV infection (Jariyapan et al., 2018; Leelayoova et al., 2017a). For instance, research conducted in southern Thailand observed a prevalence rate of 25.1 % among HIV patients (Manomat et al., 2017a). The study used multivariate logistic regression analysis to show that individuals living in stilt houses had a higher risk of acquiring the infection compared to those in non-stilt houses (OR = 1.60). Moreover, participants with CD4+ levels between 200 and 500 cells/μL (OR = 2.13) or below 200 cells/μL (OR = 1.98) were at greater risk of contracting *Leishmania* than those with CD4+ levels exceeding 500 cells/μL. Additionally, the analysis indicated that non-injection drug abusers were more likely to present *Leishmania* seropositivity than non-abusers (OR = 2.23). Similarly, participants with CD4+ levels between 200 and 500 cells/μL had a higher likelihood of being seropositive compared to those with levels above 500 cells/μL (OR = 2.09). Lastly, individuals with a detectable viral load >50 copies/mL faced an increased risk of having detectable *Leishmania* DNA in their blood (OR = 2.31) than those with an undetectable viral load after accounting for the mentioned variables.

As previously noted (Liautaud et al., 2015), VL and CL can occur when *Leishmania* and HIV infection coexist (Chusri et al., 2012). Table 2 shows some studies conducted on patients infected with L. *martiniquensis*. Table 3 shows similar studies regarding L. *martiniquensis* infection with animal hosts.

**Table 2:** Review of clinical data on leishmaniasis caused by *Leishmania martiniquensis.*

Author (year)	Area	Gender	Age (years)	HIV status	CD4+ T cell count (cells/mm3)	Primary clinical presentation	Clinical manifestations	Samples	Treatment
Chusri et al., 2012	Songkhla, southern Thailand	Male	46	Positive	175	VL	Marked pallor and petechiae, a single painless, well-defined, and punched-out ulcer surrounded by erythematous plaque with serous oozing and granulation tissue on top 3 × 3 cm, left groin lymphadenopathy, anemia and thrombocytopenia	Bone marrow, discharge from the ulcer, urine and oral fluid	AmB, ITZ.
	Trang, southern Thailand	Male	30	Positive	111	VL	Multiple papules, single ulcerative lesion, anemia, abdominal distention pale, liver enlargement, mild splenomegaly, infiltrative papules and plaques with ulcers, granulation tissue	Bone marrow, papule biopsies, ulcer biopsies, oral fluid	AmB, ITZ
Pothirat et al., 2014	Chiang Mai, Northern Thailand	Male	52	Negative		VL	Low grade fever, fatigue, weight loss, moderate pallor without jaundice, huge splenomegaly	Bone marrow	AmB
Chiewchanvit et al., 2015	Chiang Mai, northern Thailand	Male	48	Positive	121	CL, VL	Multiple discrete firm hypopigmented and brownish papules and nodules on his inner canthi, eyelids, nose, helices and antihelices of both pinnae, and extensor surfaces of hands, forearms and legs and hepatosplenomegaly	Blood, skin, bone marrow	AmB, ITZ
	Lumphun, northern Thailand	Male	38	Positive	543	CL, VL	Brownish papules on the dorsum of both hands, the lesions became enlarged and extended to both elbows, legs both palms and his face, intermittent fevers, multiple discrete hypopigmented papules and nodules at the inner and outer canthi of eyes, helices and antihelices of both pinnae, and extensor surfaces of hands, forearms and legs	Blood, skin bone marrow	AmB, ITZ
Liautaud et al., 2015	Caribbean	Male	61	Positive	186	VL	Hypertension, Hepatosplenomegaly, anemia, and permanent fatigue	Bone marrow, whole blood	AmB
Songumpai et al., 2022	Sadao, Songkhla, southern Thailand	Female	46	Negative		VL	Prolonged fever, myalgia, fatigue, significant weight loss for 2 months, body temperature of 37.8 °C, mild pallor and splenomegaly	Bone marrow	AmB, ITZ
	Sadao, Songkhla, southern Thailand	Male	51	Positive	26	VL	Prolonged fever, anorexia, fatigue, weight loss of 10 kg in 2 months, body temperature of 38.3 °C, marked pallor, hepatosplenomegaly, persistent high grade fever, voluminous and diarrhea	Bone marrow, whole blood, saliva	AmB, ITZ
Srivarasat et al., 2022	Chiang Rai, northern Thailand	Female	47	Positive	185	DCL, MCL, VL	Multiple ulcerated papules, nodules, and plaques all over the face and body, multiple firm nodules on the hard palate mucosa and nasal opening, hepatosplenomegaly and bilateral lymphadenopathy on both sides of her groins	Bone marrow, saliva, cutaneous nodular biopsy, whole blood	AmB, ITZ

CL = cutaneous Leishmaniasis; VL = visceral Leishmaniasis; DCL = diffused cutaneous Leishmaniasis; MCL = mucocutaneous Leishmaniasis; Amphotericin B = AmB; Itraconazole = ITZ.

**Table 3:** Review of animal data on leishmaniasis caused by *Leishmania martiniquensis.*

Reference	Location	Animal	Clinical presentation	Clinical manifestations	Samples
Menezes et al., 2019	Florida, USA	Horse	CL	Bilateral, non-healing wounds on the ear pinnae	Skin biopsy
Mendes Junior et al., 2023	Rio de Janeiro State, Brazil	Horse	CL	Multiple cutaneous lesions in the left pinna with a history of difficult healing	Skin biopsy
Lobsiger et al., 2010	Switzerland	Cow	CL	Several ulcerative or plaque-like skin lesions 1–10 cm on the muzzle, bilateral carpal regions, ear bases, the udder and the thoracic wall, and a very large oval-shaped ulcerated cutaneous mass 20 × 5 × 4 cm on the ventral abdomen	Cutaneous mass
Müller et al., 2009	Germany, Switzerland	Horse	CL	Skin nodules, cutaneous lesions	Skin biopsy Immunohistochemistry PCR

CL = cutaneous leishmaniasis.

### 1.6 Treatment

Early detection of the disease and timely implementation of efficient treatment decrease its prevalence and protect patients from serious consequences (Egamov Xasan, 2021). Amphotericin B deoxycholate (AmB) is the primary medication used to treat leishmaniasis, a disease caused by parasites. AmB works by binding to sterols, which are important components of the parasites' cell membranes. Several studies employed this medication for the treatment of L. *martiniquensis*. Studies used AmB at a dosage of 1 mg/kg/day for the treatment (Songumpai et al., 2022a). Nevertheless, the vulnerability of L. *martiniquensis* to AmB wasn't previously examined, in addition to, there have been instances of disease recurrence following treatment in certain cases in patients who were not immunocompromised although they were seronegative, such as HIV positive. This suggests the need for enhanced chemotherapy (Leelayoova et al., 2017a; Osatakul et al., 2014).

It is possible that the therapeutic benefits and advantages of using combinations of various medications and/or chemicals outweigh those of using any one of these substances alone. In addition, low solubility, poor absorption, renal toxicity, other significant side effects such as fever, thrombophlebitis, and chills with rigidity are drawbacks of current AmB therapy (Laniado-Laborin and Cabrales-Vargas, 2009). There has been restricted clinical usage of AmB due to the long-term injectable dosing, which frequently necessitates hospitalized (Sundar and Chakravarty, 2015). Natural compounds with anti-leishmanial potential include Allicin and andrographolide, both of which are easily available. Allicin has demonstrated anti-leishmanial activity against the amastigotes stages of L. *donovani* and L. *infantum,* without causing significant effects on mammalian cells (Corral-Caridad et al., 2012; Corral et al., 2014). It has also shown inhibitory effects on the growth of *L. mexicana* and L. *infantum* promastigotes in laboratory settings (McClure et al., 1995). Allicin exhibits a synergistic effect with AmB in combating intracellular amastigotes of L. *donovani* and *L. infantum*, resulting in a two-fold decrease in AmB dosage (Corral et al., 2014). Additionally, it has demonstrated the ability to prevent the development of L. *major* promastigotes (Metwally et al., 2016). Corral et al. demonstrated that the combination of allicin and AmB exhibited a variety of synergistic effects, varying from highly synergistic to low concentrations. For instance, when 0.07 μM AmB was combined with 35.45 μM allicin, it resulted in a remarkable 95 % inhibition of growth (Corral et al., 2014). Additionally, andrographolide showed potent anti-leishmanial activity against macrophages infected with L. *donovani* in vivo, which is an intriguing attribute to consider (Sinha et al., 2000). There have been reports of it having anti-trypanosomal activity (Banerjee et al., 2017) and anti-plasmodial activity against erythrocytic stages (Mishra et al., 2011).

Intakhan et al. carried out an investigation to evaluate the susceptibility of L. *martiniquensis* to Allicin and andrographolide, regarding the goal of improving treatment options for L. *martiniquensis* contamination by reducing the negative impacts of AmB. The study also looked at what happened when Allicin or andrographolide was mixed with AmB on intracellular amastigotes in PEMs, focusing on how they worked together. The findings demonstrated that L. *martiniquensis* exhibited a high susceptibility to AmB. However, both Allicin and andrographolide displayed selectivity index (SI) values exceeding 10, suggesting potential efficacy of these drugs in treating host cells infected with L. *martiniquensis*. There were no apparent synergistic effects when combining AmB and andrographolide (Intakhan et al., 2020).

In 2021, Chanmol et al. (Chanmol et al., 2022) conducted a study assessing the anti-leishmanial activity of 8-hydroxyquinoline (8HQN) and its synergistic effects with AmB against L. *martiniquensis* in vitro. The results indicated that 8HQN exhibited significant anti-*Leishmania* activity against L. *martiniquensis*, with IC50 values of 1.60 ± 0.28 μg/mL for promastigotes and 1.56 ± 0.02 μg/mL for intracellular amastigotes. Furthermore, the combination of 8HQN and AmB showed synergistic effects in combating intracellular amastigotes without causing harm to host cells. These findings suggest that a combination therapy approach involving 8HQN and AmB could be a viable option for treating infections caused by this *Leishmania* spp., and may serve as an alternative treatment for leishmaniasis. 8HQN works as a chelating agent to decrease RNA synthesis in some yeast and bacteria such as *Schizosaccharomyces pombe* yeast and also *Clostridium difficile* (Fraser and Creanor, 1974; Novakova et al., 2014). 8HQN destroyed intracellular infections such *Cryptococcus neoformans* and *Mycobacterium tuberculosis* by activating Cu-independent host responses (Festa et al., 2014; Shah et al., 2016). 8HQN did not trigger the host response to eliminate parasites because it did not promote macrophage production of nitric oxide (Chanmol et al., 2022). However, Duarte et al. (Duarte et al., 2016) demonstrated that 8HQN does not cause oxidative stress. Instead, it specifically targets the mitochondria of the parasite, influencing the parasite's mitochondrial membrane potential and ultimately eradicating the parasite. Another interesting finding is that 8HQN had no effect on the permeability of the parasites' plasma membranes. This goes against the way AmB works, which is to lyse the parasite cells by binding to ergosterols (Mishra et al., 2007).

Furthermore, as the Table 2 illustrates, studies have employed various treatments or combinations thereof, including: Triamcinolone acetonide, and itraconazole (Chusri et al., 2012; Ryan et al., 2006).

## 2 Conclusion

Our data demonstrated that L. *martiniquensis* infections pose a significant public health concern, particularly in individuals with compromised immune systems, such as those living with HIV/AIDS. In general, *L. martiniquensis* appears to be an emerging and globally distributed *Leishmania* species capable of causing both cutaneous and visceral disease in humans, with important implications for diagnosis and treatment of leishmaniasis in affected regions. Southeast Asian countries experience higher levels of these infections. The male gender had the highest rate of infection, with an average age of 46.5 years. The clinical manifestations of L. *martiniquensis* infections can vary widely, ranging from CL to VL. However, VL was the most common type of clinical presentation. Risk factors for the infection include the presence of domestic animals, age, gender, and environmental factors. Furthermore, investigations have shown L. *martiniquensis* in black rats, demonstrating its ability to spread from humans and animals to various animal species. This parasite has also been reported in other animals, such as horses and cows. The main treatment for the infection is AmB deoxycholate, which can be combined with other agents to reduce side effects. However, further research is needed to develop more effective treatments and prevent the spread of the disease. Global and regional health organizations play a crucial role in providing public information and improving health facilities to prevent the disease and its effects. As whole, the future research priorities for L. *martiniquensis* include elucidating its full geographic distribution, understanding the determinants of its diverse clinical manifestations, optimizing treatment approaches, identifying competent vector species, and developing robust diagnostic tools to enhance surveillance and case detection.

## Ethics approval and consent to participate

Not applicable.

## Funding

No specific fund received for this work.

## CRediT authorship contribution statement

**Somayyeh Ahmadi:** Writing – review & editing, Writing – original draft, Resources, Investigation. **Maryam Hataminejad:** Writing – review & editing, Methodology, Data curation. **Bahman Rahimi Esboei:** Writing – review & editing, Methodology, Investigation, Data curation. **Seyed Abdollah Hosseini:** Writing – review & editing, Methodology, Investigation. **Mahdi Fakhar:** Writing – review & editing, Writing – original draft, Supervision, Methodology, Investigation, Data curation, Conceptualization.

## Declaration of competing interest

There is no conflict of interest.
